# Type II Toxin–Antitoxin Systems in *Pseudomonas aeruginosa*

**DOI:** 10.3390/toxins15020164

**Published:** 2023-02-17

**Authors:** Meng Li, Nannan Guo, Gaoyu Song, Yi Huang, Lecheng Wang, Yani Zhang, Tietao Wang

**Affiliations:** Provincial Key Laboratory of Biotechnology, Key Laboratory of Resource Biology and Biotechnology in Western China, Ministry of Education, College of Life Sciences, Northwest University, Xi’an 710069, China

**Keywords:** *Pseudomonas aeruginosa*, type II, toxin–antitoxin system, biological function, regulatory mechanism

## Abstract

Toxin–antitoxin (TA) systems are typically composed of a stable toxin and a labile antitoxin; the latter counteracts the toxicity of the former under suitable conditions. TA systems are classified into eight types based on the nature and molecular modes of action of the antitoxin component so far. The 10 pairs of TA systems discovered and experimentally characterised in *Pseudomonas aeruginosa* are type II TA systems. Type II TA systems have various physiological functions, such as virulence and biofilm formation, protection host against antibiotics, persistence, plasmid maintenance, and prophage production. Here, we review the type II TA systems of *P. aeruginosa*, focusing on their biological functions and regulatory mechanisms, providing potential applications for the novel drug design.

## 1. Introduction

Toxin–antitoxin (TA) systems were first discovered on a conjugative plasmid in 1983, functioning as plasmid maintenance systems by post-segregational killing (PSK) [1]. They are small genetic modules composed of a stable toxin and a labile cognate antitoxin. Currently, eight types of TA systems (types I–VIII) are classified based on the nature of the antitoxin and the molecular mode of action. The toxins of all known TA systems are proteins, with the exception of type VIII toxins (RNAs); the antitoxins are RNA or protein [2]. In type I, III, and VIII TA systems, antitoxins are RNAs, and type I and III antitoxins bind to toxin mRNAs and proteins, thereby inhibiting toxin expression and toxicity, respectively [2]. Type VIII antitoxins and toxins are RNAs that bind to each other to repress RNA toxin expression [3]. The type II, IV, V, VI, and VII TA system toxins and antitoxins are proteins. Antitoxin proteins block the toxicity of the toxins via the following mechanisms: direct protein–protein interactions (type II) [4,5,6], compete with and remove the toxin from its target (type IV) [7,8], cleave the toxin mRNA (type V) [9], promote toxin degradation (type VI) [10], or regulate post-translational chemical modifications (type VII) [11,12].

The toxin inhibits an essential cellular process (kills cells or inhibits their growth), while the antitoxin counteracts the toxicity of its cognate toxin [2,13]. TA systems are widespread in bacterial and archaeal chromosomes and mobile genetic elements (plasmids, prophages, transposons, and integrate and conjugate elements) and have diverse roles in bacterial physiology and pathogenicity [2]. The plasmid-encoded PrpT/PrpA TA system controls the plasmid copy number; these new insights improve the understanding of the TA systems in the role of bacterial physiology [14]. Among all types of TA systems, type II TA systems are highly prevalent in bacterial genomes and have been evaluated extensively. They are implicated in the maintenance of genetic material, virulence and pathogenesis, biofilm formation, phage inhibition, and stress response [15,16].

*Pseudomonas aeruginosa* is one of the ESKAPE pathogens (*Enterococcus faecium*, *Staphylococcus aureus*, *Klebsiella pneumoniae*, *Acinetobacter baumannii*, *Pseudomonas aeruginosa*, and *Enterobacter* sp.) and is a critical priority pathogen [17]. Dai et al. found 16,963 TA gene hits in 5432 genomic sequences of *P. aeruginosa* from the NCBI Genome database, most of which were in chromosome sequences (94%) and belonged to type II TA systems (16,808 hits) [18]. To date, at least 10 pairs of type II TA systems have been experimentally characterised in *P. aeruginosa*, 8 of which are located on the chromosome [19,20,21,22,23,24,25,26], whereas PumA/PumB and PfiT/PfiA were identified in plasmid pUM505 and prophage Pf4, respectively [27,28]. These TA systems have multiple biological functions and regulatory mechanisms, which makes TA research more interesting and attractive. Here, we summarise recent findings on the functions and regulatory mechanism of type II TA systems in *P. aeruginosa*.

## 2. Transcriptional Regulation of Type II TA Systems in *P. aeruginosa*

**Figure 1:**
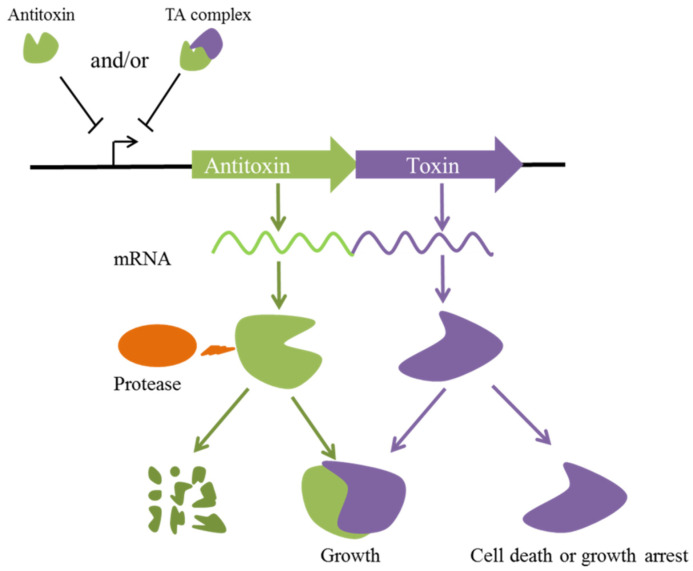
Model of type II toxin–antitoxin systems. The toxin and antitoxin are represented in purple and green, respectively. The antitoxin protein directly interacts with cognate toxin protein and inhibits its toxicity. The labile antitoxin is efficiently degraded when the production of protease, the stable toxin, leads to cell death or growth arrest. The antitoxin and/or toxin–antitoxin (TA) complex can negatively autoregulate the transcription of their operators by recognizing and binding to palindromic sequences.

Ten pairs of type II TA systems have been experimentally verified in *P. aeruginosa* (Table 1), as follows: HigB/HigA [19], ParD/ParE [20], HicA/HicB [21], RelB/RelE [22], ResA/XreA [23], PrrT/PrrA [24], CrlT/CrlA [25], PacT/PacA [26], PumA/PumB [27], and PfiT/PfiA [28]. In type II TA systems, genes encoding the toxin and antitoxin are within a single operon (Figure 1). In the *pfiTA*, *parDE*, *resA/xreA*, *prrTA*, *crlTA*, and *relBE* TA systems, the antitoxin gene precedes the toxin gene, and the whole operon is transcribed by a single promoter. By contrast, in the *higBA*, *hicAB*, *pumAB*, and *pacTA* TA systems, the toxin gene is upstream of the antitoxin gene; of the two promoters, the first promoter is responsible for the expression of the whole operon, and the second promoter controls the expression of the antitoxin. TA system expression is tightly autoregulated at the transcriptional level [6]. Antitoxins are the critical regulator and typically comprise a DNA-binding domain that recognises and binds to palindromic sequences in the promoter regions of the cognate TA operon [6]. The antitoxin alone or the TA complex as a transcriptional repressor autoregulates TA operon expression [29]. The *higBA*, *crlTA*, *prrTA*, and *resA/xreA* TA system toxins are negatively regulated by the corresponding antitoxins. In the *pfiTA* TA system, *pfiT* and *pfiA* are co-transcribed, and the PfiTA complex binds to the palindrome sequence (5′-AATTCN_5_GTTAA-3′) overlapping the −35 region of the *pfiTA* promoter, thereby repressing the expression of the *pfiTA* operon [28]. Interestingly, the *relBE* operon is regulated by both the antitoxin RelB and the TA complex RelBE; antitoxin RelB is a weak repressor, whereas RelE is an efficient co-repressor to further increase transcriptional regulation [30,31]. However, the transcriptional regulatory mechanisms of the *pumAB*, *pacTA*, *parDE*, and *hicAB* TA systems in *P. aeruginosa* are unclear. The stoichiometries of TA complexes depend on the toxin/antitoxin ratio and affect the affinity for the operon. In the presence of excess antitoxin relative to a toxin, the operon is weakly repressed. However, excess toxin leads to the formation of saturated TA complexes, which cannot bind operons [6,32,33].

## 3. Biological Functions of Type II TA Systems in *P. aeruginosa*

**Figure 2:**
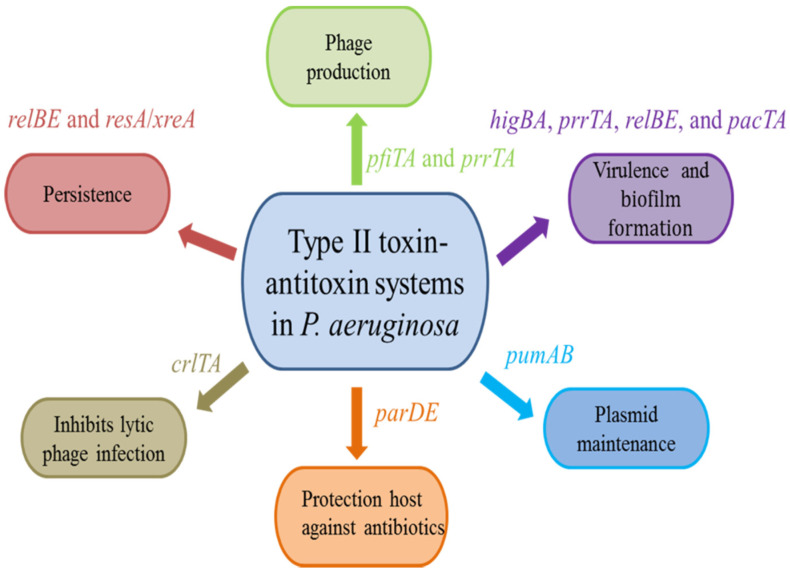
Biological functions of type II TA systems in *P. aeruginosa*.

In *P. aeruginosa*, most type II TA systems are encoded on the chromosome [19,20,21,22,23,24,25,26], the exceptions being PfiT/PfiA and PumA/PumB (phage and plasmid, respectively) [27,28]. These TA systems have diverse biological functions, such as virulence and biofilm formation [34,35,36], protection host against antibiotics [20], persistence [23], plasmid maintenance [27], and prophage production [25,37] (Figure 2).

### 3.1. Virulence and Biofilm Formation

Type II TA systems are involved in the virulence and biofilm formation of bacteria. Many chronic infections with pathogenic bacteria are associated with biofilm formation [38]. Biofilms cause chronic infection because it protects bacteria against a wide range of antimicrobial agents and enhances bacteria’s adaptable ability to survive in different environments [39,40]. The cumulative evidence suggests that TA systems are involved in biofilm formation in *P. aeruginosa* [41,42].

**Figure 3:**
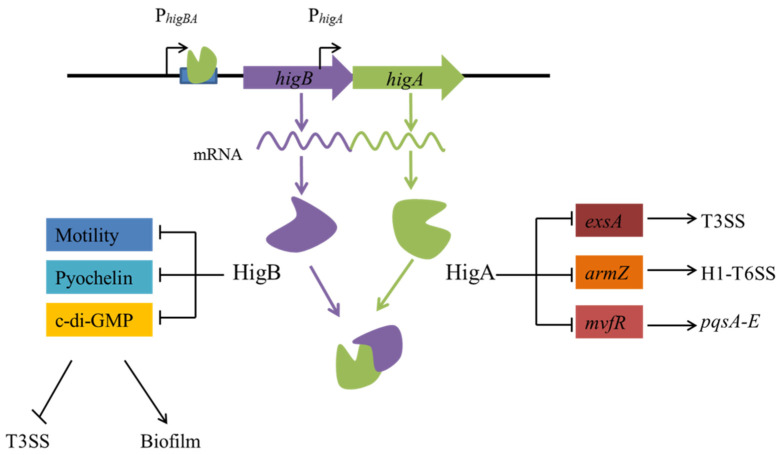
Regulatory pathways of HigB/HigA in control of virulence in *P. aeruginosa*. HigA negatively regulates the transcription of the TA operon by binding to the palindromic sequence and also negatively regulates the expression of *exsA*, *armZ*, and *mvfR* by binding to their promoter regions. HigB can repress biofilm formation and increase expression of the T3SS genes by negatively regulating the level of c-di-GMP and also negatively regulating motility and pyochelin. ‘→’ indicates induction, and ‘┤’ indicates repression.

HigBA is prevalent in *P. aeruginosa* clinical isolates and was the first TA system characterised in this species [19,22]. The toxin HigB regulates multiple virulence factors, such as pyocyanin, pyochelin, swarming motility, and biofilm formation [19] (Figure 3). The *higA* operon is induced by treatment with ciprofloxacin. HigB is involved in ciprofloxacin-induced the formation of persister cell and activates the expression of type III secretion system (T3SS) genes [43]. HigB modulates the expression of the T3SS genes and biofilm formation by regulating the expression of cyclic di-GMP (c-di-GMP) hydrolysis genes [35]. The antitoxin HigA functions as a transcriptional repressor of *mvfR*, *exsA*, and *amrZ* by directly binding to their promoter regions, and it controls pyocyanin synthesis, T3SS, and type VI secretion system (T6SS) expression [34,44]. *mvfR* is a key virulence-related regulator of *P. aeruginosa* that activates the expression of the *phnAB* and *pqsA-E* operons [45]. *exsA* is the master regulator that activates the expression of all the T3SS genes [46]. *amrZ* is a global T6SS transcriptional regulator that activates H1-T6SS and H3-T6SS but represses H2-T6SS expression by binding to the promoter regions of T6SS genes [47].

The RelBE TA system was discovered in the chromosome of *Escherichia coli* K-12 [48,49], and *relBE* genes were detected in 100% of *P. aeruginosa* [50]. RelBE TA systems are associated with biofilm formation, antibiotic resistance, oxidative stress, and persistence [51]. *relBE* expression is increased in isolates that are sensitive, compared with isolates that are resistant to aztreonam [50]. Zadeh et al. showed that the expression of *relBE* promotes persister cell formation in biofilms in the presence of ciprofloxacin and colistin in *P. aeruginosa* [52]. RelBE TA systems control biofilm formation by indirectly regulating the expression of biofilm-associated genes [36].

The PrrTA TA system is encoded by the chromosome of the clinical *P. aeruginosa* isolate 39016, 3504 bp upstream of prophage *att* sites [24]. Deletion of *prrA* significantly increased biofilm formation and reduced motility [24]. The *amrZ* transcript level was significantly decreased in a Δ*prrA* mutant, a global regulator of genes associated with a virulence that indirectly modulates the c-di-GMP level by repressing diguanylate cyclase genes or activating phosphodiesterase genes [53,54]. Moreover, AmrZ regulates bacterial motility and alginate synthesis [55]. In addition, the *prrTA* system is involved in prophage regulation and production. Most prophage genes are significantly downregulated in the Δ*prrA* mutant, and phage production and infectivity are significantly lower than those in the wild type [24].

The novel type II TA system PacTA has been characterised by *P. aeruginosa* PA14. The acetyltransferase toxin PacT inhibits the DNA-binding activity of Fur (ferric uptake regulator) to maintain iron homeostasis by directly binding to the HTH domain of Fur [26]. Fur is a central transcriptional repressor that modulates iron uptake processes by repressing the expression of genes responsible for iron acquisition and storage [56]. Moreover, PacTA contributes to pyocyanin biosynthesis and biofilm formation in *P. aeruginosa*. Compared with the wild-type, pyocyanin production and biofilm formation were markedly decreased in the Δ*pacT* and Δ*pacTA* mutants [26]. Fur represses biofilm formation in pathogens such as *Yersinia pestis* [57] and *Stenotrophomonas maltophilia* [58]; PacTA may contribute to biofilm formation by the path. PacTA also contributes to the virulence of *P. aeruginosa*; compared with the wild type, the Δ*pacT* and Δ*pacTA* mutants caused lower mortality.

### 3.2. Protection Host against Antibiotics

The ParDE TA system was characterised in plasmid RK2 as a plasmid stabilisation element [59]. This TA system is distributed in the IncI and IncF-type antibiotic resistance and virulence plasmids in *E. coli* and *Salmonella* species [60]. ParDE confers a survival advantage to the host under antibiotic and other stress conditions [60]. In *P. aeruginosa*, the toxin ParE promoted survival in the presence of quinolone antibiotics, e.g., ciprofloxacin, levofloxacin, and novobiocin [20]. However, higher concentrations of ParE decrease cell viability and alter cell morphology by inhibiting DNA gyrase [20]. ParDE is the only TA system capable of switching from a protective to a toxic effect depending on the toxin concentration [20]. This is the first example that the TA system can exert either protective or toxic effects, depending on the amount of toxin present. Nine chromosomal ParE toxins from various bacterial species alter cellular morphology from rods to filaments, consistent with disruption of DNA topology [61]. This phenotype is a marker of ParE toxin activity.

### 3.3. Persistence

Persister cells are slow-growing or growth-arrested cells that can resume growth after the stress disappears [62]. Persister formation is related to antibiotic tolerance and to refractory and chronic infections. Persistence is associated with TA systems, and TA systems play a major role in persistence formation [63]. The ResA/XreA (PA14_51010/PA14_51020) TA system was discovered in *P. aeruginosa* strain PA14. The toxin ResA belongs to the RES family, and the antitoxin XreA is a xenobiotic response element (Xre) [23]. ResA/XreA affects bacterial survival in the presence of tobramycin and ciprofloxacin [23]. Overexpression of ResA promotes persistence and reduces the intracellular NAD^+^ level, a key component of the respiratory machinery. Therefore, a reduced intracellular NAD^+^ level could increase antibiotic resistance. Overproduction of NAD^+^ counteracts the effect of overexpression of ResA on persister formation, suggesting that ResA/XreA contributes to persister formation by reducing the intracellular NAD^+^ level [23]. However, the deletion of *resA* did not affect persister formation due to additional TA systems or other determinants involved in persister formation.

### 3.4. Plasmid Maintenance

Plasmid maintenance is a function of plasmid-encoded type II TA systems [15]. The two earliest identified TA systems, *ccdAB* and *hok-sok*, stabilise plasmids by PSK [1,64]. PSK systems are addiction systems that encode long-lived toxins and short-lived antitoxins, ensuring plasmid stability by killing plasmid-free daughter cells [15]. The functions of plasmid-encoded TA systems in *P. aeruginosa* are unclear. The PumAB TA system is encoded from the conjugative plasmid pUM505 of *P. aeruginosa* strain PUM503 [27]. pUM505 harbours a mercury resistance operon (*merRTPFADE*), chromate resistance operon (*chrBAC*), and ciprofloxacin resistance gene (*crpP*) [65]. Non-*pumAB*-carrying plasmids decreased in number after 216 generations and were undetected after 432 generations; by contrast, *pumAB*-carrying plasmids were not significantly affected [27]. Therefore, *pumAB* can confer post-segregational plasmid stability via PSK [65]. No mechanism other than PSK has been confirmed in *P. aeruginosa.* Additionally, the PumA toxin confers *P. aeruginosa* virulence and increased Caenorhabditis *elegans* and mouse mortality rates [66].

### 3.5. Phage Production

Prophages and satellite prophages are widely distributed among bacteria, in which they confer various phenotypic traits to their hosts, including pathogenicity [67], antibiotic tolerance [68], biofilm formation [41], and general stress [41]. Prophage-borne TA systems have been discovered in *E. coli* [69] and *Shewanella oneidensis* [70]. TA systems are also found to be responsible for controlling the production of prophages. The PfiTA TA system is encoded by the filamentous Pf4 prophage of the *P. aeruginosa* PAO1 strain [28]. The Pf4 prophage modulates physiology and virulence, affects biofilm matrix composition and structure, and enhances bacterial survival and antibiotic resistance [71,72]. Deletion of the toxin gene *pfiT* increased Pf4 phage production by approximately 100,000-fold compared with the wild type. In the ∆*pfiT* mutant, the phage replication initiation gene *PA0727* was induced but not the phage repressor gene *pf4r*. Therefore, PfiT regulates Pf4 phage production by inducing the expression of the replication initiation gene *PA0727* [28]. In addition, the toxin *PfiT* is involved in phage immunity and coordinates the phage repressor Pf4r in conferring immunity to Pf4 [28]. The chromosome-encoded CrlTA TA system in *P. aeruginosa* WK172 is involved in fighting against phage infection. In excess, the prophage Cro-like antitoxin CrlA inhibited infection by lytic *Pseudomonas* phages (PAP-L5, PAOP5, PAP8, and QDWS) because CrlA inhibited phage replication by binding to *crlTA* palindrome-like sequences in the phage genome [25]. The CrlTA complex confers phage resistance to lytic phages PAP8 and QDWS; however, the underlying molecular mechanism is unclear.

## 4. Potential Application of Type II TA Systems in *P. aeruginosa*

Bacterial TA systems have considerable biotechnological potential [2,73,74]. For instance, a novel antibiotic that targets specific pathogens can be developed based on toxin stability. Moreover, antitoxin or TA complexes could repress virulence gene expression, protect against stresses, and inhibit phage infection. TA systems have much potential for the development of novel antibacterials [73,74,75,76,77,78].

TA systems can be used as positive selectable markers for ensuring the stability of mobile genetic materials [2]. An example is TA systems as positive selection vectors. A toxin gene in such a vector will be inactivated by the insertion of a segment of foreign DNA; therefore, only bacteria harbouring the recombinant vector will proliferate [1]. Another example is plasmid stabilisation. The antitoxin gene, under the control of a constitutive promoter, is cloned into a plasmid. The toxin gene under the control of a promoter strongly repressed by the antitoxin protein is cloned into the bacterial chromosome. Therefore, a bacterial cell lacking the vector will not survive. This allows plasmid stabilisation without antibiotics [79].

## 5. Concluding Remarks

Although TA systems have been widely distributed and well studied in pathogenic bacteria for decades, in *P. aeruginosa*, the biological functions of TA systems have been focused on recently. Ten pairs of type II TA systems have been identified in *P. aeruginosa*. In *P. aeruginosa*, type II TA systems enhance fitness and have multiple biological functions. For instance, the HigBA TA system is associated with virulence and biofilm formation, persister formation, T3SS, and T6SS. Interestingly, the ParDE TA system confers protective or toxic effects depending on the toxin concentration, but the molecular basis remains unknown for the switch from protective effects at low concentrations to toxic effects at higher concentrations. The HicAB TA system has known functions in other bacteria, e.g., virulence, persister cell formation, and stress responses [80,81]; however, the HicAB system has no known function in *P. aeruginosa* [21].

The antitoxin can autoregulate TA operon transcription and also regulate other key regulatory genes. The antitoxin is a negative regulator and participates in repressing the expression of other key regulatory genes [82,83,84]. In *P. aeruginosa*, the antitoxin HigA regulates the transcriptional regulatory genes such as *mvfR* [44], *exsA* [34], and *amrZ* [34], which are involved in regulating virulence expression, T3SS, and T6SS, respectively. More novel type II TA systems are found in clinical isolates; however, their roles in clinical environments are unclear. Further investigations should focus on the medical applications of TA systems.

## Figures and Tables

**Table 1:** Type II toxin–antitoxin systems identified and characterised in *P. aeruginosa*.

TA System	Toxin	Antitoxin	Localisation	Targeted Cellular Process	Function
HigB/HigA	HigB	HigA	chromosome	Translation	Virulence and biofilm formation
PumA/PumB	PumA	PumB	plasmid	Unknown	Virulence and Plasmid maintenance
PfiA/PfiT	PfiT	PfiA	prophage	Unknown	Phage production and immunity
ParD/ParE	ParE	ParD	chromosome	Replication	Protection host against antibiotics
HicA/HicB	HicA	HicB	chromosome	Translation	Unknown
RelB/RelE	RelE	RelB	chromosome	Translation	Persistence and biofilm formation
ResA/XreA	ResA	XreA	chromosome	Metabolic stress	Persistence
PrrA/PrrT	PrrT	PrrA	chromosome	Unknown	Phage production and biofilm formation
CrlA/CrlT	CrlT	CrlA	chromosome	Translation	Inhibits lytic phage infection
PacA/PacT	PacT	PacA	chromosome	Translation	Virulence and biofilm formation

## Data Availability

Not applicable.
